# Characteristics of the Fungal Communities and Co-occurrence Networks in Hazelnut Tree Root Endospheres and Rhizosphere Soil

**DOI:** 10.3389/fpls.2021.749871

**Published:** 2021-12-08

**Authors:** Wenxu Ma, Zhen Yang, Lisong Liang, Qinghua Ma, Guixi Wang, Tiantian Zhao

**Affiliations:** ^1^Key Laboratory Tree Breeding and Cultivation of the National Forestry and Grassland Administration/Research Institute of Forestry, Chinese Academy of Forestry, Beijing, China; ^2^State Key Laboratory of Tree Genetics and Breeding, Beijing, China; ^3^National Hazelnut Industry Innovation Alliance of National Forestry and Grassland Administration, Beijing, China; ^4^Hazelnut Engineering and Technical Research Center of National Forestry and Grassland Administration, Beijing, China

**Keywords:** hazelnut, fungal community, co-occurrence network, roots, surrounding soil

## Abstract

Hazelnut has gained economic value in China in recent years, but its large-scale planting and research started later than other countries. Conducting basic research on hazelnut trees requires studying their related microorganisms. Here, we used high-throughput DNA sequencing to quantify the fungal communities in the root endospheres and rhizosphere soil of four hazelnut species. Fungal diversity in the rhizosphere soil was significantly higher than that in the root endospheres. Rhizosphere soil had more Mortierellomycota, and the fungal community compositions differed among the four hazelnut species. The root endospheres, especially those of the Ping’ou (*Corylus heterophylla* × *Corylus avellana*) trees, contained more ectomycorrhizal fungi. The co-occurrence networks in the rhizosphere soil were more sophisticated and stable than those in the root endospheres, even when the root endospheres had higher modularity, because the structural differentiation of the root endospheres differed from that of the rhizosphere soil. Two-factor correlation network analysis and linear regression analysis showed that the total organic carbon was the main environmental factor affecting the fungal communities. Our study revealed the community compositions, functional predictions, and co-occurrence network structural characteristics of fungi in hazelnut root endospheres and rhizosphere soil. We also examined the potential keystone taxa, and analyzed the environmental factors of the dominant fungal community compositions. This study provides guidance for the growth of hazelnut and the management of hazelnut garden, and provides an insight for future development of fungal inoculants to be used in hazelnut root.

## Introduction

Hazelnut trees are one of four nut trees in the worldwide that provide nuts with high nutritional and economic value. Turkey is a major producer of hazelnuts, its hazelnuts are mainly *Corylus avellana*. China’s hazelnut industry started in the early 1980s, with the cross-breeding of *Corylus heterophylla* and *C. avellana*, and new varieties were cultivated until the early 21st century. In 2016, the planting area of *C. heterophylla* × *C. avellana* reached over 50,000 ha and continues to increase rapidly (Wang, 2018; Wang et al., 2018). China is an important origin of *Corylus*, with eight native species which are widely distributed in 22 provinces (municipalities and autonomous regions) in northeast, north, northwest and southwest China, with *C. heterophylla* and *Corylus kweichowensis* being the most widely distributed (Zhang et al., 2005).

Fungi are an indispensable part of the microbial system and played an important role in ecosystems (Van der Wal et al., 2013; Ning et al., 2020). The research on rhizosphere microorganisms is one of the frontier and hot spots in plant science research in recent years. Studies have shown that rhizosphere microbial community played a significant role in determining the growth and health of plants in soil-plant system (Liu et al., 2019). In the soil, the root system of plants is not only an organ for fixing plants and absorbing water and nutrients, but also a place for microorganisms to gather, inhabit and multiply. These root microorganisms accompany the whole growth cycle of plants, and help them to absorb nutrients, resist diseases, and adapt to stress environment (Oldroyd et al., 2011; Bulgarelli et al., 2013; Santhanam et al., 2015; Mbodj et al., 2018). In addition, rhizosphere microorganisms can compete with host plants for nutrients in soil, or attack plants as pathogenic microorganisms (Berendsen et al., 2012). Endophytic microorganisms can colonize plant species without causing any diseases (Petrini, 1991). Previous studies have explored the community composition of rhizosphere microorganisms and root endophytes in *Mussaenda kwangtungensis*, Cacti, bean, and poplar, which provided a way to understand the relationship between soil and plants (Fonseca-García et al., 2016; Beckers et al., 2017; Qian et al., 2019; da Silva et al., 2020). Exploring the relationship between plants and the microorganisms in their environment can increase the understanding and utilization of these microorganisms, which is helpful to improve the productivity and economic value of plants (Waller et al., 2005). Previous studies on hazelnut microorganisms mainly focused on using ectomycorrhiza to promote hazelnut growth (Román et al., 2006; Wedén et al., 2009; Santelices and Palfner, 2010; Benucci et al., 2012), however, there are few reports on the composition of microbial community and the relationship of root endospheres and rhizosphere microorganisms in hazelnut species. The study on fungi in root endospheres and rhizosphere soil is helpful to understand the interaction between rhizosphere fungi and plants, and to screen potential growth-promoting fungi which are beneficial to plant growth.

Proulx et al. (2005) first put forth the idea of co-occurrence and networks in ecology, and these terms are widely used in soil and plant microbial ecology. Although network analysis has some problems (Faust and Raes, 2012), it is important for revealing interactions among microbial community members, the symbiotic modes of microorganisms in plants and soil, and the responses of microbial communities to environmental changes that cannot be determined by conventional microbial community analysis (Barberán et al., 2012; de Vries et al., 2018; Fan et al., 2018; Banerjee et al., 2019; Qian et al., 2019; Tu et al., 2020). Studies have shown that the disturbance of protective microorganisms in rhizosphere can promote the occurrence of diseases, stable and complex microbial community plays an important role in plants under drought and other stresses (Lee et al., 2020; Gargouri et al., 2021). The network of a healthy tree may need to be stable to maintain protective effects. Additionally, microbial networks exhibit modularity, an important ecological concept. Network modularity refers to the degree to which species interactions are organized into modules. Modularity can reflect the heterogeneity of habits and the selective mechanisms of differentiation (Olesen et al., 2007). Deng et al. (2012) used several methods to define modules and submodules within a large module and considered that the greedy modularity optimization approach better identified the submodular structure of molecular ecological networks in microbial communities. Module hubs and connectors are keystone taxa in a network, which are highly related taxonomic groups according the greedy modularity optimization approach. Keystone species play a unique and key role in the microbial community. Their removal will change the structure and function of the microbial community, and then affect the ecology of its ecosystem (Bardgett and Van Der Putten, 2014; Fierer, 2017; Banerjee et al., 2018).

Here, we studied the compositions of fungal community, and revealed the differences of fungal functions between the root endospheres and rhizosphere soil of four hazelnut tree species. We also evaluated the stability of the fungal co-occurrence network and explored the potential keystone taxa in the root endospheres and rhizosphere soil. Finally, we explored the dominant environmental factors influencing fungal community formation. This study may provide theoretical guidance for hazelnut growth, managing hazelnut garden, and provides an insight for future development of fungal inoculants to be used in hazelnut root.

## Materials and Methods

### Study Area and Sampling

The study area was located in the experimental station of Yanqing District, Beijing, China. The annual average temperature is 8°C and daylight lasts 2800 h annually. According to a wet-sieving fractionation method first described by Cambardella and Elliott (1993), the macroaggregates (>0.25 mm), free microaggregates (0.25–0.053 mm) and non-aggregated silt + clay fractions (<0.053 mm) were obtained. The percentages of macroaggregates (>0.25 mm) free microaggregates (0.25–0.053 mm) and non-aggregated silt + clay fractions (<0.053 mm) were 28.04, 26.75, and 45.21%. *C*. *heterophylla* (PZ), *C*. *kweichowensis* (CZ), *Corylus avellane* (OZ), and *C*. *heterophylla* × *C*. *avellane* (ZJ) were planted in 2014 in the same growth state, then managed in the same way every year. Row spacing for each tree was 1.5 m × 2 m. Three randomly arranged plots of 10 m × 10 m were constructed for each species in April of 2019. We used sterile gloves for sampling. The roots of *Corylus* are relatively developed, so in order to keep the original living state of microorganisms in the roots and rhizosphere soil, we took back the roots and the surrounding soil together. When sampling, we used shovels and scissors treated with 70% ethanol. Soil and roots of six trees in four directions were taken from each plot. To avoid cross infection, spades and scissors were disinfected before sampling in each direction. The soil and roots of the six trees obtained were taken as a repetition, and three such samples were taken for each species. After all samples were refrigerated and transported to the laboratory as soon as possible, the soil attached to the root was shaken off, and the remaining soil that remained closely attached to the root was called rhizosphere soil (Fan et al., 2018). The shaken soil was screened using a 2-mm sieve to determine the physical and chemical properties of the soil, and the root samples were placed into 50 mL sterile tube, and 10 mm phosphate buffered saline (PBS) buffer (130 mM NaCl, 7 mM Na_2_HPO_4_, 3 mM NaH_2_PO_4_, and pH 7.4), shake washing twice, taking out the roots, putting them into a 50 mL sterile tube, adding 10 mM PBS, washing for 10 min by ultrasonic wave (160W, 30/30 s), finally collecting the buffer solution of three times, centrifuging 13,000 g for 10 min, and collecting the precipitate. The washed roots were washed with sterile water, then soaked in 70% ethanol for 2 min, then soaked in 2.5% NaClO for 5 min, then transferred to 70% sterile ethanol for 30 s, and finally the roots were washed with sterile water for three times. To verify the effectiveness of surface disinfection, roots were placed in a Petri dish containing maltose (MEA, 2%) and cultured in the dark at 25°C for 48 h to check the appearance of colonies.

### Soil Physicochemical Properties

The soil pH was measured by pH meter (Mettler-Toledo, S40 SevenMulti™, Greifensee, Switzerland) with a 2.5:1 ratio of water to soil (Qian et al., 2015). The soil water content (SWC) was determined as described by the Institute of Soil Science Chinese Academy of Sciences (1978). The total organic carbon (TOC) content was determined *via* the K_2_CrO_4_ oxidation method, the total nitrogen (TN) content was measured by the Kjeldahl method, and the total phosphorus (TP) content was measured *via* the NaOH alkali fusion-atomic absorption method. Available phosphorus (AP) was determined using the Olsen method, and available potassium (AK) was measured using a flame photometer after NH_4_OAc extraction (Qian et al., 2014).

### DNA Extraction and Sequencing

Microbial DNA was extracted from the samples using the E.Z.N.A.^®^ Soil DNA Kit (Omega Bio-tek, Norcross, GA, United States) per the manufacturer’s instructions. The ITS sequence was amplified with the primers ITS1F (5′-CTTGGTCATTTAGAGGAAGTAA-3′) and ITS2R (5′-GCTGCGTTCTTCATCGATGC-3′) (Bulgarelli et al., 2015). PCRs were performed in triplicate in 20-μL reactions containing 4 μL of 5 × FastPfu Buffer, 2 μL of 2.5 mM dNTPs, 0.8 μL each primer (5 μM), 0.4 μL FastPfu Polymerase, and 10 ng template DNA. The amplification process consisted of an initial denaturation at 95°C for 2 min, followed by 25 cycles at 95°C for 30 s, 55°C for 30 s, and 72°C for 30 s, and a final extension at 72°C for 5 min. Amplicons were extracted from 2% agarose gels and purified using the AxyPrep DNA Gel Extraction Kit (Axygen Biosciences, Union City, CA, United States) according to the manufacturer’s instructions and were quantified using QuantiFluor™-ST (Promega, United States). The NEXTflex™ Rapid DNA-Seq Kit (Bioo Scientific, United States) was used to build the database. The steps of building the database are divided into four steps: (1) linker linking; (2) using magnetic beads to screen and remove the linker self-connected fragments; (3) enriching the library template by PCR amplification; and (4) recovering PCR products by magnetic beads to obtain a final library. Sequencing was carried out by using Miseq PE300 platform of Illumina Company. The fastp software (version 0.20.0^1^) was used for quality control of the original sequencing sequence, and the FLASH software (version 1.2.7^2^) was used for splicing. According to the similarity of 97%, UPARSE (version 7.1^3^) was used to check chimera sequences. The taxonomy of each operational taxonomic unit (OTU) representative sequence was analyzed by RDP Classifier version 2.2 against the ITS database (unite 8.0) using confidence threshold of 0.7. Rarefaction was used to calculate diversity indices of data. All sequence data were deposited in the NCBI Sequence Read Archive (SRA) database under accession number SRP323811 and BioProject ID PRJNA737048.

### Statistical Analyses

Homogenization is carried out according to the minimum number of sample sequences to keep the number of all sample sequences consistent. Statistical analyses of the OTU richness, Shannon diversity, evenness, and good’s coverage indexes were performed in Mothur (version 1.30.1). To assess the significance of the differences in fungal diversity in the root endospheres and rhizosphere soil, Wilcoxon rank-sum test, and Kruskal-Wallis H test were performed using the “stats” package in R (version 3.3.1) to conduct two groups of difference tests (Figures 2C, D) and multiple groups of difference tests (Supplementary Figure 2), respectively. Principal co-ordinates analysis based on Bray–Curtis-faith distance algorithm was used to analyze the difference of fungal composition between root endospheres and rhizosphere soil. Adonis analysis was performed using the ‘‘vegan’’ package in R (version 3.3.1) to analyze the explanatory degree of different grouping factors to the differences of samples, and substitution test was used to analyze the statistical significance of the division. Fungal community functions were classified and analyzed using FUNGuild.^4^ The fungi in the analysis were the species that belong to a single guild (Zhou et al., 2020). Trophic mode were divided into three types: pathotroph, symbiotroph, and saprotroph. To reduce complexity, only abundant OTUs with total read proportions >0.005% were used in the OTU table (He et al., 2017), and the co-occurrence networks were made by Gephi (version 0.9.2^5^). The greedy modular optimization method was used to detect modules (Deng et al., 2012). Node attributes of the topology were divided into four types based on their within-module (Zi) and among-module (Pi) connectivity values: module hubs (center point of the module, nodes with high connectivity inside the module, Zi > 2.5 and Pi < 0.62), connectors (connecting nodes, nodes with high connectivity between two modules, Zi < 2.5 and Pi > 0.62), network connectors (network center points, nodes with high connectivity in the whole network, Zi > 2.5 and Pi > 0.62) and Peripherals (peripheral nodes, nodes without high connectivity within and between modules, Zi < 2.5 and Pi < 0.62). Module hubs, connectors and network connectors were classified as key nodes (Guimerà and Amaral, 2005; Olesen et al., 2007; Deng et al., 2012).

## Results

### Fungal Community Diversity, Compositions, and Function

**FIGURE 1:**
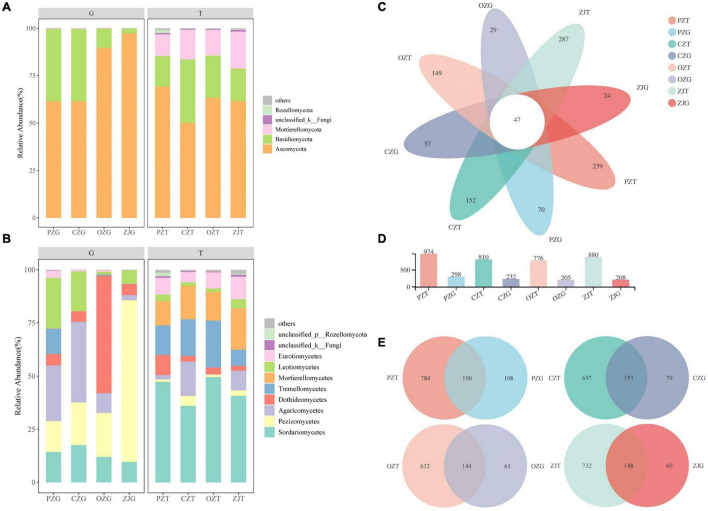
Fungal composition of the rhizosphere soil (T) and root endospheres (G) of hazelnut species: relative abundances of soil fungal community structure at the phyla **(A)** and class **(B)** levels; Venn diagram of core OTUs among each of hazelnut species **(C–E)**.

The dominant fungal phyla in the root endospheres were Ascomycota (77.55%) and Basidiomycota (22.17%), and the dominant fungal phyla in the rhizosphere soil were Ascomycota (61.08%), Basidiomycota (22.24%), and Mortierellomycota (15.02%). The dominant fungi in the root endospheres were Pezizomycetes (32.81%), Agaricomycetes (18.95%), and Dothideomycetes (17.63%), and the dominant fungi in the rhizosphere soil were Sordariomycetes (43.42%), Tremellomycetes (15.24%), and Mortierellomycetes (14.99%) (Figure 1A). The percentages of Agaricomycetes in the PZ and CZ root endospheres were 26.15 and 37.86%, respectively. Dothideomycetes (55.11%) was the most abundant in the OZ root endospheres, and Pezizomycetes (75.99%) was the most abundant in the ZJ root endospheres (Figure 1B). *Tuber* content in roots of all hazelnut species were higher than that in rhizosphere soil (Supplementary Figure 1). All samples contained 47 common OTUs, and the rhizosphere of each hazelnut species contained significantly more fungal OTUs than did the root endospheres (Figures 1C,D). The common OTUs of root endospheres and rhizosphere soil were 190 for PZ, 153 for CZ, 144 for OZ, and 148 for ZJ (Figure 1E). The Shannon and richness indexes showed that the fungal diversity in each sample was significantly higher in the rhizosphere soil than in the root endospheres. The coverage indexes for all samples exceeded 99% (Supplementary Table 1).

**FIGURE 2:**
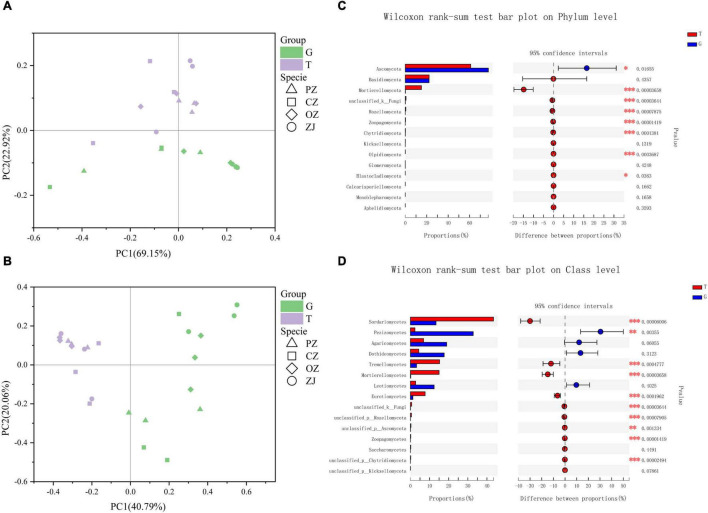
Principal co-ordinates analysis of the rhizosphere soil (T) and root endospheres (G) at the phyla level **(A)** and class level **(B)**; extended error bar plot showing the fifteen most abundant phyla and classes that had significant differences between root endospheres and rhizosphere soil **(C,D)**. Positive differences in mean relative abundance indicate phyla or classes overrepresented on the root endospheres (G), while negative differences indicate phyla or classes greater abundance in the rhizosphere soil (T). *P < 0.05; **P < 0.01; ***P < 0.001.

**FIGURE 3:**
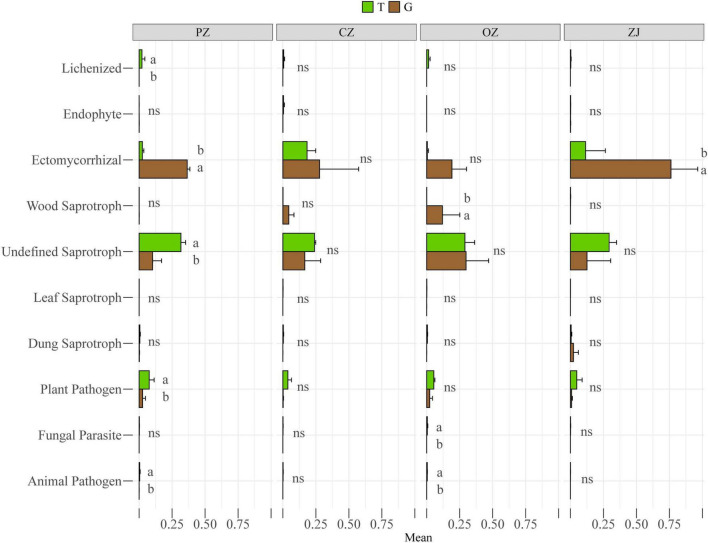
Functional features of fungal communities in four hazelnut species in the rhizosphere soil (T) and root endospheres (G). Different letters (a,b) indicate the significance level at *P* < 0.05, “ns” indicates no significance (*P* > 0.05).

Principal co-ordinates analysis (PCoA) showed that fungi in the root endospheres and rhizosphere soil could be significantly separated at the phylum and class levels, and that PC1 and PC2 could explain 92.07 and 60.85% of the differences, respectively (Figures 2A,B). Wilcoxon rank-sum tests showed that the relative content of fungi in the top 15 phyla of soil in rhizosphere was higher than that in root endospheres except ascomycetes. Mortierellomycota, unclassified_k_Fungi, Rozellomycota, Zoopagomycota, Chytridiomycota, Olpidiomycota, and Blastocladiomycota levels in the rhizosphere soil were significantly higher than those in the root endospheres. At the class level, Pezizomycetes was significantly higher than in their rhizosphere soil, whereas Sordariomycetes, Tremellomycetes, Mortierellomycetes, Eurotiomycetes, unclassified_k_Fungi, unclassified_p_Rozellomycota, unclassified_p_Ascomycota, Zoopagomycetes, and unclassified_p_Chytridiomycota were significantly higher in the rhizosphere soil than in the root endospheres. However, the root endospheres and rhizosphere soil did not significantly differ between each hazelnut species in phylum and class levels (Supplementary Figure 2). Adonis analysis showed that the explanatory degree of the plant compartments group (root endospheres and rhizosphere soil) factor to the sample difference is 0.212 (phylum) and 0.407 (class), and the explanatory degree of the species group (four hazelnut species) factor to the sample difference is 0.523 (phylum) and 0.710 (class). *P*-value were all less than 0.05, which showed that the test is highly reliable (Supplementary Figure 3). Although *R*^2^ of the species group was smaller than that of the compartments group, but the species group was more significant according to the *P*-value (0.008 and 0.024), so the difference between groups was more significant (Supplementary Figure 3). The trophic modes included symbiotrophs (lichen, endophytes, and ectomycorrhiza), saprotrophs (wood saprotrophs, undefined saprotrophs, leaf saprotrophs, and dung saprotrophs) and pathotrophs (plant pathogens, fungal parasites, and animal pathogens) (Figure 3 and Supplementary Table 2). The prediction results of the fungal functional guild revealed that ectomycorrhiza were the main symbiotrophs. Ectomycorrhizal abundances were significantly higher in the PZ and ZJ root endospheres than in the rhizosphere soil.

### Network Analysis of Fungal Communities

**FIGURE 4:**
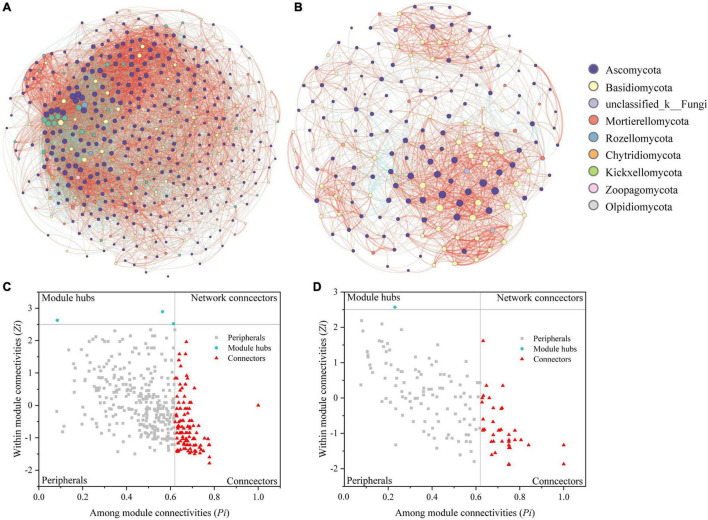
Networks of fungal communities in the rhizosphere soil **(A)** and root endospheres **(B)**. Node color represents fungal phylum of the OTUs. Node size is proportional to degree connections of the OTUs. Red edges indicate positive relationships, and green edges indicate negative relationships. Zi-Pi plots showing distribution of OTUs based on their topological roles in the rhizosphere soil **(C)** and root endospheres **(D)** networks. Threshold values of Zi and Pi for categorizing OTUs were 2.5 and 0.62, respectively.

We constructed correlation networks of the fungal communities in the root endospheres and rhizosphere soil and obtained two networks of 460 and 184 points connected by 8091 and 1629 edges, respectively (Figures 4A,B and Supplementary Table 3). The rhizosphere soil contained more nodes and edges than the root endospheres. The networks of the root endospheres and their rhizosphere soil showed more positive correlations than negative correlations, at 1.93 and 7.44 times higher, respectively. Compared with the network structure of the root endospheres, the network structure in the rhizosphere soil was more connected (connectivity) and less modular (modularity) (Supplementary Table 3).

Modular analysis revealed 122 connectors and three module hubs in the rhizosphere soil and 49 connectors and one module hub in the root endospheres. The root endospheres and rhizosphere soil contained nine common connector OTUs. The module hubs in the rhizosphere soil were OTU1819, OTU2475 (*Chloridium*) and OTU3920 (*Acaulium*); the module hub in the root endospheres was OTU1769 (*unclassified_f__Melanommataceae*) (Supplementary Tables 4, 5). The fungal functions of the connectors and module hubs were mainly symbiotrophic and saprotrophic. No network hubs were detected in the two networks (Pi > 0.62, Zi > 2.5; Figures 4C,D and Supplementary Tables 4, 5).

### Relationships Among Soil Properties and Fungal Communities

**FIGURE 5:**
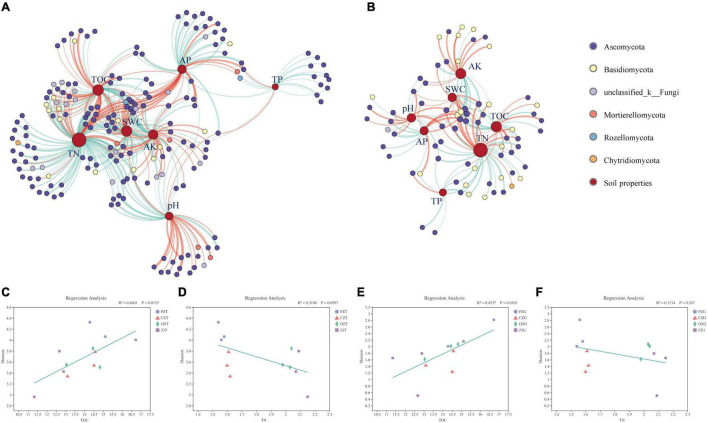
Two-factor correlation network between soil properties and fungi OTUs (**A**: the rhizosphere soil, **B**: the root endospheres). Node color represents fungal phylum of the OTU. Node size is proportional to degree connections of the OTU. Red edges indicate positive relationships, and green edges indicate negative relationships. The linear regression analysis of environmental factors was based on the results of principal coordinates analysis **(C–F)**. X and Y axes are the Shannon diversity of fungi and the environmental factor, and R2 is the determination coefficient, representing the proportion of variation explained by regression line.

At the OTU level, the correlation network between fungal OTUs and environmental factors (Figure 5 and Supplementary Table 7) in the rhizosphere soil surrounding the roots was more complex than that of the root endospheres, consisting of 189 and 71 OTUs, respectively (Supplementary Tables 7–10). Among the fungal OTUs in the rhizosphere soil surrounding the roots, Ascomycota and unclassified_k_Fungi accounted for the highest proportions, at 76.53 and 8.67%, respectively. Ascomycota and Basidiomycota accounted for 62.82 and 25.64%, respectively, of the fungal OTUs in the root endospheres. TN and TOC were the soil properties with the highest degrees in the two networks. Linear regression results showed that the Shannon diversity index of the fungi in the root endospheres and rhizosphere soil had a significant positive linear relationship with TOC (*P* = 0.0153, *P* = 0.0163, respectively) and a non-significant negative linear relationship with TN.

## Discussion

Our results showed that fungal diversity and community compositions differed between hazelnut tree root endospheres and rhizosphere soil (Figures 1, 2). Similar results have been found for the microbial compositions of poplar trees, *Mussaenda kwangtungensis* and rice (Beckers et al., 2017; Edwards et al., 2018; Qian et al., 2019). This may be because root exudates, mucus produced by the root cap and detached root cells provide suitable niches for the microbial communities around roots (Buée et al., 2009). Ascomycota and Basidiomycota were the dominant fungal phyla in the root endospheres and rhizosphere soil, which was consistent with previous reports (Ma et al., 2013; da Silva et al., 2020; Hou et al., 2020). Ascomycota is the most abundant phylum in the rhizosphere community (Qian et al., 2019; Gargouri et al., 2021), the dominant phylum in the soil of larch plantation, and the main decomposer in many ecosystems (Wang et al., 2013, 2016; Zhou et al., 2016). Basidiomycota can produce lignin-modifying enzymes and is considered a decomposer under natural conditions (Blackwood et al., 2007). Mortierellomycota, formerly classified as Zygomycota, is an indicator of rhizosphere soil (Anslan et al., 2018; Geml, 2018), which was confirmed by the results of the current study. Function prediction results (Supplementary Table 2) showed that the main functions of Mortierellomycetes were symbiotrophic and saprotrophic (Cannon and Kirk, 2007). At the class and genus level, PZ and CZ root endospheres contained high proportions of Agaricomycetes, and the function prediction results showed that most Agaricomycetes were ectomycorrhizal fungi and other symbiotic fungi, which were consistent with the results of previous studies that showed that many Agaricomycetes were more ectomycorrhiza (Chen et al., 2006; Zeng and Mallik, 2006; Kersten and Cullen, 2013). The dominant fungal classes in the ZJ root endospheres was Pezizomycetes, whose fungal functions were predicted to be symbiotrophic and saprotrophic (Supplementary Table 2). Among these, the dominant species were *Tuber*, which are edible fungi with important nutritional and economic value. Previous studies have shown that *Tuber* can improve the rooting rate and root lengths of hazelnut trees (Román et al., 2006; Wedén et al., 2009; Santelices and Palfner, 2010; Benucci et al., 2012). These *Tuber* characteristics provide guidance for improving the survival rates of hazelnut seedlings and the economic output of hazelnut orchards. FUNGuild prediction results (Figure 3) revealed more symbiotic fungi (mainly ectomycorrhizal fungi) and fewer pathogenic fungi in the root endospheres than in the rhizosphere soil, possibly because most fungi in the root endospheres were beneficial microorganisms recruited by plants from the rhizosphere soil through the interface between the roots and soil. Most of these fungi live in healthy plant tissues or organs and do not cause the host plants to show disease symptoms (Petrini, 1991; Liu et al., 2021; Song et al., 2021; Yin et al., 2021). The Venn diagram and intergroup difference test (Figures 1C–E, 2C,D) revealed that fungi in the root endospheres came mostly from the rhizosphere soil, supporting the conclusion that induction factors of roots can attract fungi to colonize the roots (Edwards et al., 2015). There were many ectomycorrhizal fungi in ZJ, which can be considered as isolation materials for further development of ectomycorrhizal fungi suitable for hazelnut growth and development.

Co-occurrence analysis enables understanding the interactions between plant fungal communities (Shi et al., 2016; Xue et al., 2018; Gargouri et al., 2021; Hernandez et al., 2021). This study revealed that root endospheres and rhizosphere soil have different fungal co-occurrence network structures, which can be explained by their different microenvironments. The network in the rhizosphere soil had more nodes and edges, higher community diversity, higher connectivity, and a more complex network structure. These characteristics are thought of as the representation of complex and stable network structure (Hernandez et al., 2021). High modularity was also an indicator of the network structural stability. However, in this study, the modularity of the root endospheres was higher than that of rhizosphere soil, and the clustering coefficient of the root endospheres network was higher than that of the rhizosphere soil, possibly owing to the higher differentiation degree of the root tissues (cortex and vascular tissue) compared with that of the rhizosphere soil. This structure separates microorganisms into different structures and may thereby reduce fungal community diversity and microorganismal interactions, which explains why the network structure of the roots is relatively simple (Qian et al., 2019). Low modularity indicated that cross-module association between taxa may be more common. If environmental disturbances affected the microbes in the rhizosphere soil, the disturbances that affected the taxa in one module would likely spread to other modules. Relatively more associations were noted among taxa in different modules in the rhizosphere soil (Hernandez et al., 2021). Strikingly, most of the relationships between the fungal communities in the root endospheres and rhizosphere soil were positive, indicating that most fungi had similar guilds or niches and were mutually beneficial rather than competitive (Deng et al., 2016; Feng et al., 2017).

Many microbial studies have focused on identifying modules in networks because modules play important roles in ecology and evolutionary biology (Olesen et al., 2007; Deng et al., 2012; Shi et al., 2016; He et al., 2017; Fan et al., 2018). We found many connectors and a few module hubs in hazelnut tree root endospheres and rhizosphere soil. Studies have shown that OTUs that are divided into module hubs and connectors may function as keystone taxa. Compared with other OTUs, these OTUs play important roles in network structure, and disappearance of these keystone taxa may cause modules and networks to be disassembled (Paine, 1995; Power et al., 1996; Faust and Raes, 2012). In this study, the rhizosphere soil contained more module hubs and connectors, indicating that the network structure of the rhizosphere soil was more stable than that of the root endospheres. Shared OTUs with unique or multiple functions coexist in diverse habitats and have highly complex and powerful communication capabilities. Additionally, some low-abundance groups play disproportionate roles in regulating ecological functions in different habitats, thus revealing the key role that some rare species play in an ecosystem (Deng et al., 2016; Yang et al., 2016; Feng et al., 2017; Zhang et al., 2018). Therefore, the nine connecting OTUs are keystone fungal taxa, and their overlapping root endospheres and rhizosphere soil should be evaluated in future studies. OTU1770, from the genus *Tuber*, provided some hypotheses for the development and use of hazelnut seedlings and hazelnut garden management. Ascomycota was the dominant phylum in the root endospheres and rhizosphere soil.

Fungal function prediction analysis (Supplementary Table 2) revealed more saprophytic fungi in the soil than in the root endospheres, likely because the soil contained more litter. Additionally, the network in the rhizosphere soil contained more pathogenic OTUs than the root endospheres. In addition to recruitment of beneficial microorganisms by plants, plant root structures may hinder the invasion of pathogenic fungi. Compared with the networks of the rhizosphere soil the root endospheres had more phyla containing ectomycorrhizal fungi. Ectomycorrhizal fungi play important roles in acquiring and transferring nitrogen, phosphorus, and potassium (Brandes et al., 1998; Jentschke et al., 2001), as indicated by the positive correlation between most OTUs in the network and nitrogen, phosphorus, and potassium indicators. TOC was significantly positively correlated with the Shannon indexes of fungi in the root endospheres and the rhizosphere soil. TN had more degrees but was not significantly correlated with the Shannon index of the fungi, thus supporting the conclusion that soil microorganisms were mainly limited by carbon rather than by nitrogen (Ekblad and Nordgren, 2002; Soong et al., 2020). Orchard use of soils can deplete their soil organic matter content, which has adverse effects on plant growth and yield. Studies have proved that the application of hazelnut husks by using biological techniques can improve the organic matter content, soil enzyme activity, and microbial biomass in hazelnut garden (Irmak, 2019). It was confirmed that living mulching treatment of hazelnut orchard could increase soil organic matter content and microbial diversity (Ma et al., 2021), and similar conclusions were reached in apple orchard and crop field in previous studies (Chen et al., 2014; Qian et al., 2015; Schmidt et al., 2019). Therefore, applying mulch containing organic matter to the soil surface can be a management measure of hazelnut garden.

This study revealed significant differences in the fungal community compositions and co-occurrence networks in hazelnut tree root endospheres and the rhizosphere soil. However, fungal community compositions in the leaf endospheres, phyllospheres, and stems of hazelnut trees and their relationships are also important and will be studied in our future work. In summary, our research revealed that keystone taxa in the fungal communities of root endospheres and rhizosphere soil can be developed and used as beneficial microbial communities, thus helping improve the survival rates of hazelnut seedlings and the economic income from hazelnut orchards, providing theoretical guidance for managing hazelnut orchards, and providing an insight for future development of fungal inoculants to be used in hazelnut root.

## Data Availability Statement

The datasets presented in this study can be found in online repositories. The names of the repository/repositories and accession number(s) can be found in the article/Supplementary Material.

## Author Contributions

WM carried out the experiments, collected and organized the data, and wrote the manuscript. ZY, QM, and LL participated in the data analysis, reviewed the manuscript, and gave constructive suggestions. GW participated in the design experiment and guided the research. TZ put forward the basic hypothesis of this work, designed the experiments, and helped organize the structure of manuscript. All authors read and approved the final manuscript.

## Conflict of Interest

The authors declare that the research was conducted in the absence of any commercial or financial relationships that could be construed as a potential conflict of interest.

## Publisher’s Note

All claims expressed in this article are solely those of the authors and do not necessarily represent those of their affiliated organizations, or those of the publisher, the editors and the reviewers. Any product that may be evaluated in this article, or claim that may be made by its manufacturer, is not guaranteed or endorsed by the publisher.

## Footnotes

1
https://github.com/OpenGene/fastp

2
http://www.cbcb.umd.edu/software/flash

3
http://drive5.com/uparse/

4
http://www.funguild.org/

5
https://gephi.org/users/download/

## Supplementary Material

Supplementary Figure 1 Relative abundances of fungal community structure at the genus level.

Supplementary Figure 2 Extended error bar plot showing the fifteen most abundant phyla and classes that had significant differences between four hazelnuts.

Supplementary Figure 3 Adonis analysis of plant compartments **(A,B)** and four hazelnut species **(C,D)**. **(A)** Distance calculated on phylum level of the rhizosphere soil and root endospheres. **(B)** Distance calculated on class level of the rhizosphere soil and root endospheres. **(C)** Distance calculated on phylum level of four hazelnut species in the rhizosphere soil and root endospheres. **(D)** Distance calculated on class level of four hazelnut species in the rhizosphere soil and root endospheres.

Supplementary Table 1 Fungi community diversities indices.

Supplementary Table 2 Functional features of fungal communities in four hazelnut species.

Supplementary Table 3 Fungal network properties of in root endospheres and rhizosphere soil.

Supplementary Table 4 Detailed taxonomic information for module hubs and connectors in the root endospheres.

Supplementary Table 5 Detailed taxonomic information for module hubs and connectors in the rhizosphere soil.

Supplementary Table 6 Soil properties.

Supplementary Table 7 Correlation results of correlation matrix between fungi in the rhizosphere soil and soil properties.

Supplementary Table 8 The P value of correlation matrix between fungi in the rhizosphere soil and soil properties.

Supplementary Table 9 Correlation results of correlation matrix between fungi in the root endospheres and soil properties.

Supplementary Table 10 The P value of correlation matrix between fungi in the root endospheres and soil properties.
