# Supplementary figures and images for: Characteristics of the Fungal Communities and Co-occurrence Networks in Hazelnut Tree Root Endospheres and Rhizosphere Soil
Source: Front Plant Sci. 2021 Dec 8;12:749871. doi: 10.3389/fpls.2021.749871 (PMC8692873; doi:10.3389/fpls.2021.749871)

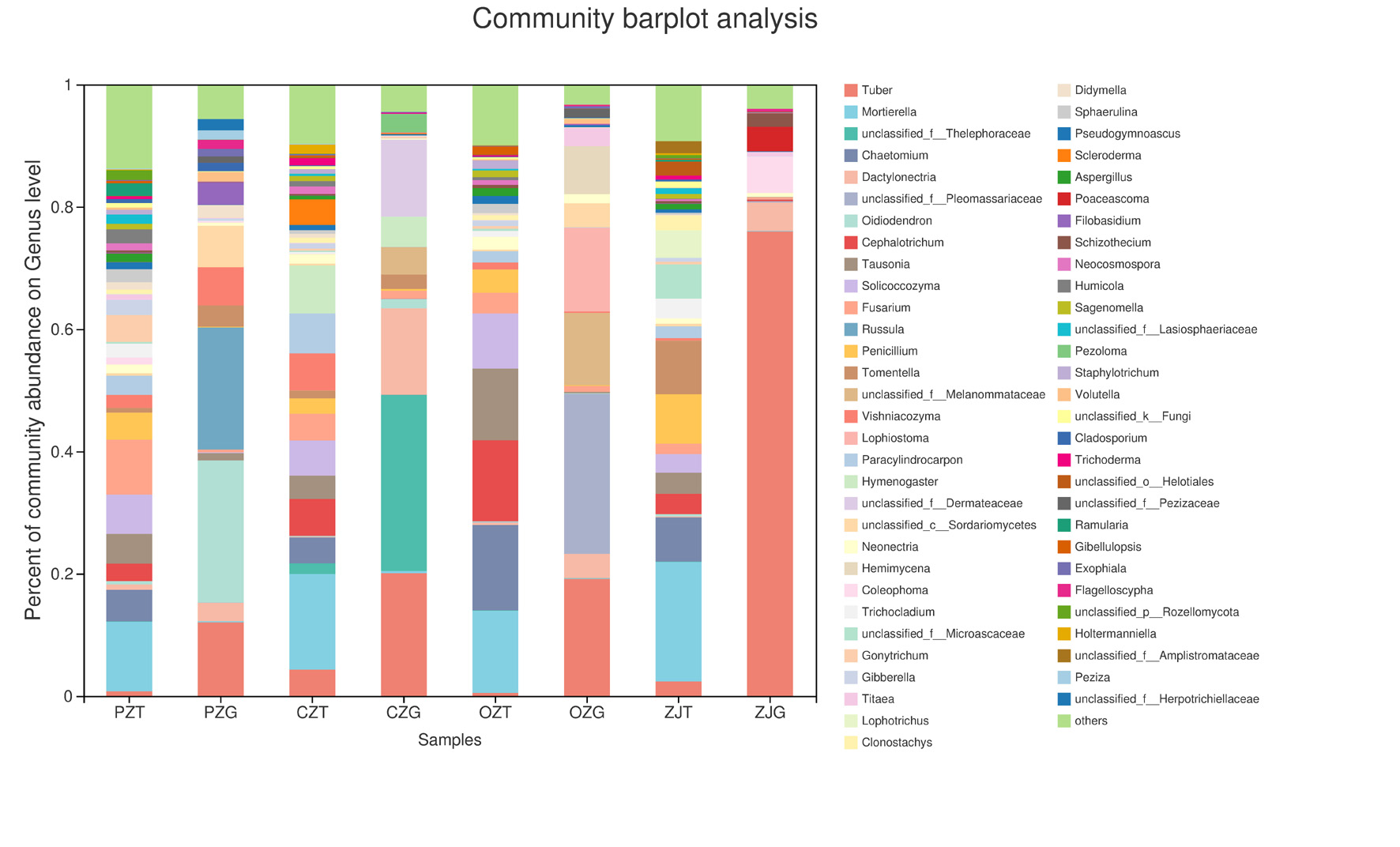

Supplement: Supplementary Figure 1 — Relative abundances of fungal community structure at the genus level. [file Image_1.JPEG]

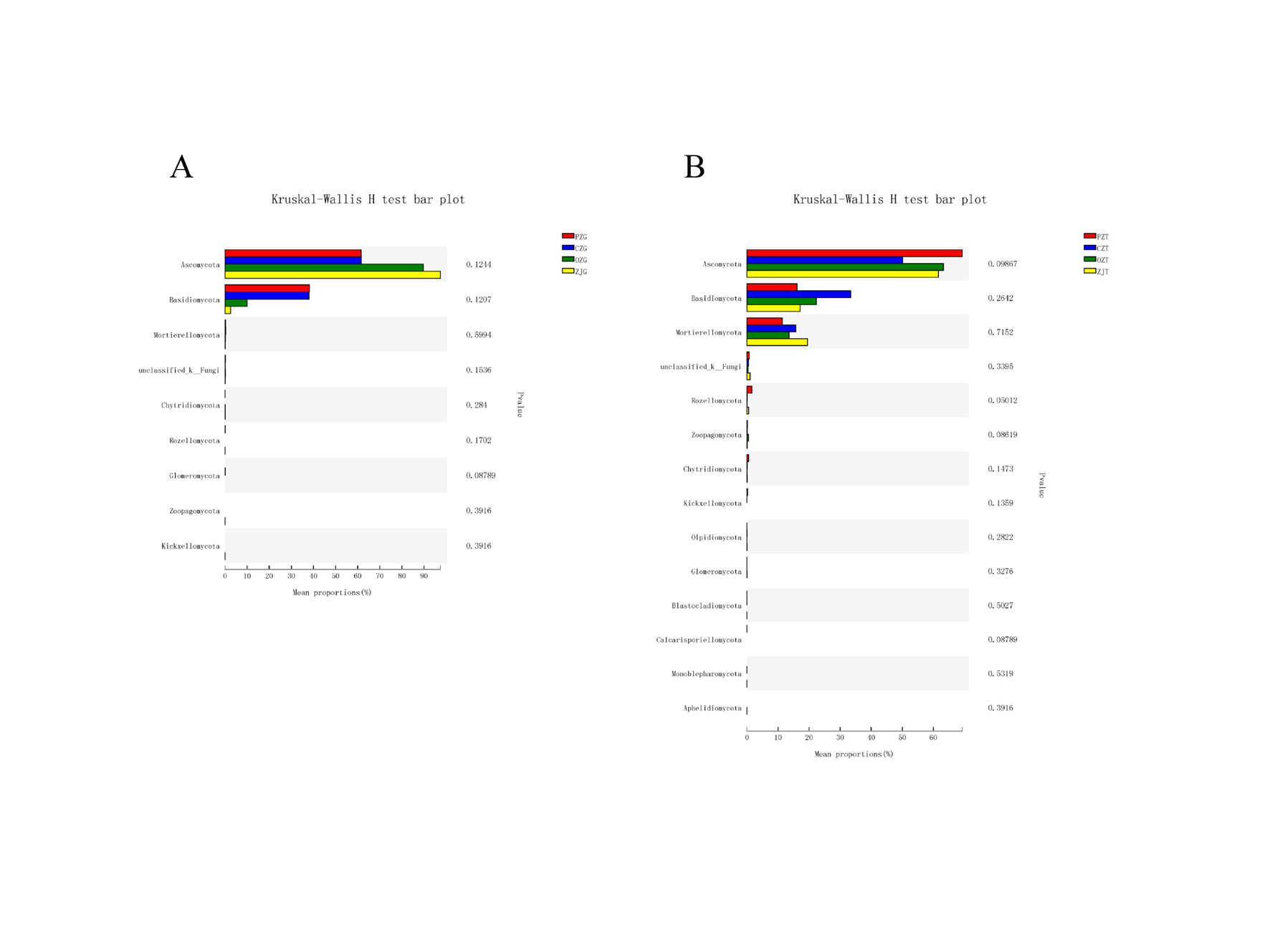

Supplement: Supplementary Figure 2 — Extended error bar plot showing the fifteen most abundant phyla and classes that had significant differences between four hazelnuts. [file Image_2.JPEG]

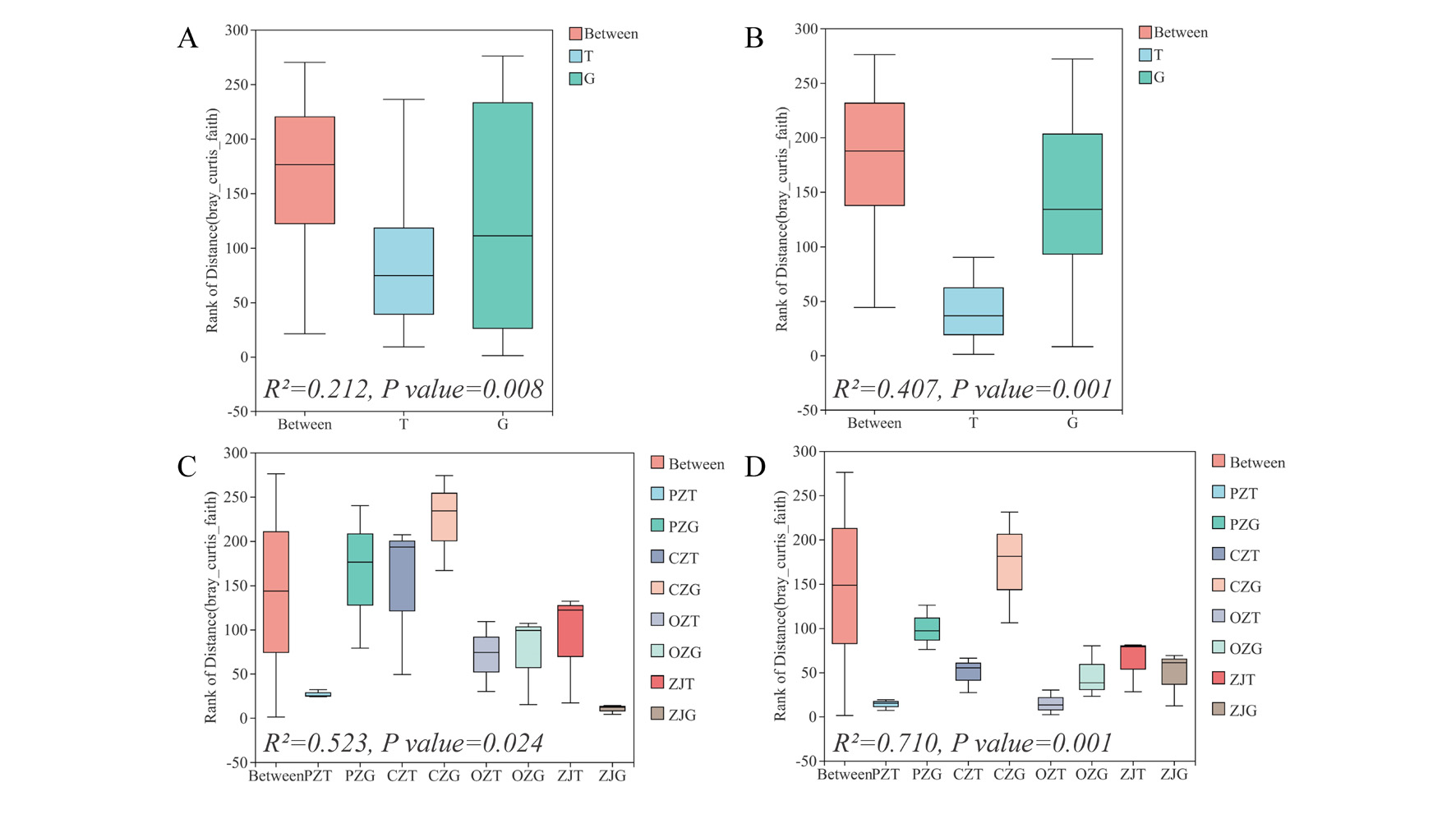

Supplement: Supplementary Figure 3 — Adonis analysis of plant compartments (A,B) and four hazelnut species (C,D). (A) Distance calculated on phylum level of the rhizosphere soil and root endospheres. (B) Distance calculated on class level of the rhizosphere soil and root endospheres. (C) Distance calculated on phylum level of four hazelnut species in the rhizosphere soil and root endospheres. (D) Distance calculated on class level of four hazelnut species in the rhizosphere soil and root endospheres. [file Image_3.JPEG]
